# Laser Therapy in Basal Cell Carcinoma: Current Evidence, Literature Gaps and Future Perspectives

**DOI:** 10.3390/bioengineering13020244

**Published:** 2026-02-20

**Authors:** Alessandro Clementi, Giovanni Cannarozzo, Luca Guarino, Luca Gargano, Martina Tolone, Elena Zappia, Marco Gratteri, Annunziata Dattola, Caterina Longo, Giovanni Pellacani, Steven Paul Nisticò

**Affiliations:** 1 Department of Clinical Sciences, Sapienza University of Rome, 00185 Rome, Italy; drcannarozzo@gmail.com (G.C.); luca.guarino@uniroma1.it (L.G.); lucagargano1995@gmail.com (L.G.); dr.martinatolone@gmail.com (M.T.); elena.zappia@hotmail.it (E.Z.); annunziata.dattola@uniroma1.it (A.D.); giovanni.pellacani@uniroma1.it (G.P.); steven.nistico@uniroma1.it (S.P.N.); 2 Department of Plastic, Reconstructive and Cosmetic Surgery, Campus Bio-Medico University Hospital, 00128 Rome, Italy; m.gratteri@unicampus.it; 3 Department of Dermatology, University of Modena and Reggio Emilia, 41124 Modena, Italy; caterina.longo@unimore.it

**Keywords:** basal cell carcinoma, laser therapy, photodynamic therapy, Nd:YAG laser, pulsed dye laser, CO_2_ laser, fractional laser

## Abstract

Basal cell carcinoma (BCC) is the most frequent skin cancer, and surgery remains the treatment of choice, particularly in high-risk subtypes and sites. However, in low-risk cases and in patients where cosmetic outcome is a priority, alternative strategies, including laser therapy, have been proposed. Different laser sources offer potential advantages in terms of minimal invasiveness, healing time, and cosmetic outcome, but their clinical role remains a matter of debate. This narrative review critically analyses the available evidence on the use of lasers in the treatment of basal cell carcinoma, with a focus on ablative lasers, vascular lasers, and laser-assisted photodynamic therapy. Mechanisms of action, main clinical results, limitations, and the emerging contribution of non-invasive imaging for case selection and response monitoring are discussed. Ablative lasers, in particular CO_2_, show favourable results in superficial low-risk BCC, while clearance reliability decreases with increasing tumour depth. Vascular lasers may offer short-term control in selected lesions but with limited long-term data. Laser-assisted PDT represents a promising strategy to extend the indication of PDT to selected nodular forms. Overall, the literature is limited by methodological heterogeneity, incomplete stratification, and short follow-ups. Well-designed comparative studies, standardised protocols, and objective controls will be essential to define the real clinical space of laser therapy in basal cell carcinoma.

## 1. Introduction

Basal cell carcinoma (BCC) is the most frequent skin neoplasm in the Caucasian population, representing about 80% of non-melanoma skin cancers [1]. The incidence is increasing, particularly among younger individuals, probably related to intermittent and cumulative sun exposure patterns [2]. The pathogenesis is associated with ultraviolet radiation and aberrant activation of the Hedgehog signalling pathway, a key driver of BCC proliferation [3,4,5].

BCC is characterised by generally slow growth and extremely low metastatic risk. However, local progression can result in significant tissue destruction, with significant functional and aesthetic impact, especially in high-risk areas of the face. In these areas, even small lesions may result in clinically significant morbidity [1,6,7].

The primary treatment management remains surgical. Standard excision, particularly Mohs micrographic surgery (MMS), guarantees the highest cure rates and represents the gold standard according to the main European guidelines [6,7,8,9]. The EADO recommendations continue to place surgery as the first-line treatment in most BCCs, with an approach stratified according to oncological risk, anatomical site, and histological subtype [6].

Non-surgical options are indicated in selected settings. Patients with multiple lesions, superficial histotypes, subjects who are not candidates for surgery, or cases where the aesthetic result is a priority require alternative strategies. In such situations, cryotherapy, curettage and electrodesiccation, photodynamic therapy (PDT), and topical treatments (e.g., imiquimod and 5-fluorouracil) represent established non-surgical approaches, often better supported by long-term evidence than emerging laser-based modalities. Importantly, non-surgical options are mainly appropriate for low-risk BCC, defined as superficial or lesions of small size (<10–15 mm), located in low-risk sites (trunk and limbs), with well-defined margins and without high-risk features (peritumoral, recurrent, or in immunosuppressed patients). High-risk BCCs, including infiltrative, morpheiform subtypes, or located in H-zones of the face, require surgical approaches with histological control of the margins [10,11,12,13,14,15,16].

In recent years, laser technologies have been proposed as a possible therapeutic option in the treatment of BCC. Ablative lasers, such as CO_2_ and Er:YAG, and sources such as Nd:YAG and pulsed dye lasers (PDL) have been employed with the aim of achieving a less invasive treatment and a potential aesthetic advantage [17,18,19,20,21]. However, at present, the role of laser remains marginal in European guidelines, with a lower level of evidence compared to methods such as cryotherapy or curettage, regarding long-term oncological control [6].

Combination protocols, especially the combination of laser and photodynamic therapy, have also been described with the aim of improving local efficacy in selected subgroups of BCC [19]. The use of CO_2_ or Er:YAG laser for superficial ablation or microperforation facilitates photosensitizer penetration, creating a synergistic effect that has shown promising results [22]. In parallel, non-invasive imaging techniques, such as in vivo confocal microscopy (RCM) and optical coherence tomography (OCT), are emerging as supportive tools for case selection and treatment monitoring [23,24,25,26,27,28].

This narrative review analyses the current role of different laser modalities in basal cell carcinoma treatment. Mechanisms of action, clinical evidence, combination protocols, and the contribution of non-invasive imaging will be discussed, with the aim of clarifying the real potential and limitations of these technologies in clinical practice. The main gaps in the literature and possible future research directions in this field will also be outlined. The bibliographic search was conducted on PubMed and Google Scholar, focusing on randomised trials, prospective studies, and clinical investigations, concentrating on laser-based and combined strategies for basal cell carcinoma. The search included articles published up to December 2025 and aimed to provide an updated perspective on laser-based approaches for BCC management. Given the narrative nature of this review, no formal methodological scoring was applied.

## 2. Laser Therapy Modalities in Basal Cell Carcinoma

Different laser sources have been investigated for the treatment of basal cell carcinoma, each targeting specific biological and structural components of the tumour. The main laser modalities and their reported clinical outcomes are summarised in Table 1 and are discussed in detail in the following subsections.

### 2.1. Ablative Lasers (CO_2_ and Er:YAG)

Ablative lasers represent one of the first laser modalities applied to the treatment of basal cell carcinoma with clinically relevant results [29]. The CO_2_ laser at 10,600 nm is the most widely used and most studied ablative source. It acts through progressive vaporisation of the tissue with control of the ablation depth. The wavelength coincides with high tissue water absorption and allows efficient energy transfer [30]. The Er:YAG laser at 2940 nm is even more selective on water. The absorption coefficient is much higher than with CO_2_, and this results in reduced ablation per pulse with less thermal collateral damage but also less coagulation capacity [31]. Most ablation protocols for BCC follow repeated passes and cleaning between passes. The goal is controlled vaporisation until a tumour-free dermal plane is achieved. The critical point remains the clearance check.

In the absence of histological control of the margins, the variable that determines the outcome is the actual depth of extension. This limitation was addressed in different ways. Horlock and colleagues ablated 51 selected BCCs with CO_2_ and then performed excision to evaluate residual tumour. The superficial ones were completely ablated if at least the middle dermis was reached. Nodulars, on the other hand, were not fully ablated reproducibly. There was a small window of safety for very small nodulars. Some nodules below 10 mm could be completely ablated if the deep dermis was reached, but this increased the risk of slow healing and scarring. This is a very useful finding because it defines a biological and technical limit of CO_2_ ablation alone when the depth increases [32]. A key limitation of ablative laser therapy is the inability to assess tumour depth before treatment. Without histological margin control, incomplete ablation becomes more likely, especially in nodular BCCs or lesions with subclinical extension. As a result, clinical clearance may overestimate histological clearance, limiting the oncologic reliability of ablative approaches to superficially confined tumours.

Another model is the intraoperative integration of cytology and histology. Campolmi et al. treated 140 patients with superficial or small nodular BCCs with superpulsed CO_2_. They performed cytological scraping before the laser, then during the procedure when the dermal papilla was recognisable, and finally when the operator considered the vaporisation to be complete. In one subset they also performed biopsies for histological control in parallel. In the follow-up reported up to 3 years, no recurrences were observed [33]. The value of the work is not only the control rate. It is the demonstration that, in experienced hands, the procedure can be set up as a step-by-step procedure with real-time biological verification. This reduces the uncertainty typical of destructive treatments.

Iyer and colleagues provided one of the longest follow-up case series on pulsed CO_2_. Retrospective study of 23 patients with 61 superficial and nodular BCCs not previously treated. The technique involved treatment of the lesion with a margin of clinically healthy skin, multiple passes, and cleansing between passes. Follow-up was extensive, up to 85 months, with an average of approximately 42 months. The clinical recurrences observed were 2 out of 61. This corresponds to about 3 per cent. Significant adverse events included one major hypertrophic scar and one case of hypopigmentation [34]. It is therefore essential to select the patient for maximum efficacy and to minimise the risk of scarring, especially when reaching deeper planes of the dermis.

Kavoussi and Ebrahimi, on the other hand, evaluated superpulsed CO_2_ in 74 patients with 113 BCCs, mostly nodular and often located on the nose. The protocol included biopsy, debulking by curettage, and 2–4 laser passes. With an average follow-up of 28 months, clearance after a single session was 93.7 per cent, with good or excellent aesthetic results in 86 per cent of cases. The authors indicate the method for small low-risk lesions and non-aggressive nodular lesions, recommending caution in larger lesions and in the nasal area [35].

Zane et al. conducted the largest available randomised trial of CO_2_ laser on superficial BCC. The study included 240 patients with trunk and limb lesions, randomised to cryotherapy, pulsed CO_2_, or surgery. Laser treatment was performed as a single session with multiple passes and peripheral margin of healthy skin. At 90 days, clinical and dermoscopic complete remission in the CO_2_ group was 78.8 per cent, lower than surgery and comparable to cryotherapy. The healing time was shorter in the laser group, with good patient satisfaction. The main limitation remains the short follow-up, which does not allow the risk of late recurrence to be assessed.

In superficial low-risk BCC, CO_2_ is effective but does not reach the robustness of surgery in terms of clearance [36]. It is important to emphasise that the reported clearance rates must be interpreted with caution, considering that most studies have limited follow-up (12–24 months), potentially insufficient to detect late relapses that may occur later than 3–5 years after treatment.

A major advance is non-invasive imaging-guided CO_2_ ablation. Navarrete and colleagues used reflectance confocal microscopy (RCM) to map low-risk BCC before and after laser treatment. In about a quarter of the cases, confocal microscopy identified residual tumour after the first pass, allowing further ablations in the same session. With a median follow-up of more than 2 years, no recurrences were observed. This approach does not replace histology but introduces lateral and deep control in real time, reducing the empirical component of laser therapy and making it more reliable in small, well-selected tumours [37]. The integration of non-invasive imaging represents a fundamental methodological advance. RCM and optical coherence tomography (OCT) allow in vivo visualisation of tumour structures before, during, and after treatment, reducing the empiricism typical of destructive approaches. RCM, in particular, can identify clinically undetectable tumour margins and neoplastic remnants after laser ablation, guiding further treatment sessions in the same session or confirming clearance without the need for immediate biopsy. This image-guided approach could become the standard for extending the use of lasers into wider clinical settings.

CO_2_ laser remains the most studied ablative source in BCC, and its most rational place is in superficial low-risk BCC and in well-selected very small nodules. The main limitation is the absence of histological margins and the difficulty of ensuring deep clearance when the phenotype is nodular. Er:YAG offers precision and less thermal damage. Its role has been studied mainly in association with photodynamic therapy [38].

Integration with non-invasive imaging represents one of the most concrete prospects for increasing reliability and reproducibility in this type of treatment.

### 2.2. Vascular Lasers (Pulsed Dye Laser and Nd:YAG)

Vascular lasers in basal cell carcinoma are based on a different rationale than ablative lasers. Instead of direct tumour vaporisation, they induce photothermolysis of the tumour-associated vascular network, leading to ischaemia and secondary damage of neoplastic tissue [39]. Pulsed dye laser (PDL) works on oxyhaemoglobin and concentrates the effect in the more superficial dermis [40,41]. This makes it more suitable for superficial BCCs and small lesions. Nd:YAG long pulse at 1064 nm penetrates deeper and can target deeper vascular structures but with less selectivity. When delivered at high fluences, both systems may also exert a direct thermal effect on tumour tissue, potentially enhancing cytotoxicity beyond the purely vascular mechanism. A clinically relevant aspect is the competing absorption of melanin at the wavelengths of vascular lasers (585–595 nm for PDL and 1064 nm for Nd:YAG). In darker phototypes (III-VI), the epidermal absorption of energy reduces the fluence available for the vascular target and increases the risk of adverse events, in particular post-inflammatory hyperpigmentation or hypopigmentation. This limits the applicability of vascular lasers in such patients and imposes a reduction in parameters, which may compromise efficacy [42,43].

Campolmi et al. described a prospective protocol on 20 patients with superficial basal cell carcinoma, often located in areas of the face, treated with five sessions of pulsed dye laser at intervals of about 3 weeks. The wavelength used was 595 nm. The parameters were within a fluence range of 6.5 to 7.5 J/cm^2^, with pulse durations between 0.5 and 1.5 ms and spot sizes of 7–10 mm, modulated according to site and clinical response. The treatment included a peripheral margin of about 5 mm and cryogen cooling, with purpuric endpoint. Sixteen out of twenty lesions showed a complete clinical response, with three recurrences observed during follow-up and one non-responder case. The value of the work is essentially practical. It defines a realistic scope of PDL use in selected superficial BCCs but confirms that recurrence remains possible in the absence of objective clearance verification [44].

In superficial low-risk BCCs, the most robust data comes from Karsai’s randomised controlled trial of 100 superficial trunk and limb BCCs. Lesions were randomised to PDL 595 nm or sham treatment. The parameters were 8 J/cm^2^, pulse duration 0.5 ms, spot 10 mm. Clinical and histological complete remission at 6 months was 78.6 per cent in the laser group versus 4.5 per cent in the sham group, with clear superiority of the active treatment. The most frequent adverse events were scabs and dyschromia, including hyper- and hypopigmentation, and about 72 per cent of patients were satisfied. This study defines a key point. PDL may be effective in the superficial cases, but the cosmetic outcome is not always predictable and should be discussed first, especially when hypopigmentation is a clinically relevant outcome [45].

Ortiz et al. reported a multicentre prospective study on Nd:YAG 1064 nm on selected non-facial BCCs. Thirty-one patients completed the protocol, which included lesions localised to the trunk and limbs smaller than approximately 2.1 cm. The treatment was performed in a single session using 5–6 mm spots, pulses of 7–10 ms, and fluence between 125 and 140 J/cm^2^, without epidermal cooling, including a 5 mm peripheral margin. Scheduled excision at 30 days documented a histological clearance of 90.3% (28 of 31 lesions), with good tolerability and no major adverse events [46].

One study evaluated a sequential combination of PDL and Nd:YAG in BCCs smaller than 2 cm, superficial, nodular or micronodular. Ten patients with 13 lesions were treated with four sessions at 2- to 4-week intervals, including the tumour and a 4 mm margin. The parameters included PDL 585 nm with 8 J/cm^2^ and 2 ms, followed by Nd:YAG 1064 nm with 40 J/cm^2^ and 15 ms, in a single pass with air cooling. The clearance was verified histologically after the last session. The overall clearance was 58%, increasing to 75% in tumours smaller than 1 cm. Tolerability was good, with no clinically relevant scarring, suggesting a possible cosmetic advantage in selected lesions [47]. However, not all studies support the efficacy of PDL in the treatment of basal cell carcinoma. Ballard et al. reported a persistence rate of 44.4% after a single treatment with PDL at 585 nm in a case series of BCC, concluding that this approach does not achieve the clearance rates achievable with standard therapeutic modalities and cannot be recommended as primary monotherapy. It is plausible that the unsatisfactory results observed are at least partly related to the use of a single session, which is insufficient to ensure adequate oncological control in the absence of histological confirmation [48]. Similarly, Chow et al. evaluated PDL at 595 nm using a double-pulse protocol in a randomised controlled trial, reporting lower cure rates than standard therapies and concluding that PDL, with the parameters adopted, cannot be recommended for the treatment of BCC [49]. In this context, the use of non-aggressive settings and limited protocols may have contributed to the negative outcomes. Overall, these data show a marked variability of results depending on the parameters and protocols used, underlining the importance of patient selection and caution when interpreting favourable results from uncontrolled studies.

Overall, vascular lasers show clinical activity in selected low-risk BCCs, mainly superficial and very small nodular lesions. Evidence remains limited by short follow-up, heterogeneous endpoints, and scarce histological confirmation. Their role therefore remains experimental and not comparable to surgery for long-term oncologic control.

### 2.3. Laser-Assisted Photodynamic Therapy

Photodynamic therapy (PDT) is a well-established treatment for superficial and low-risk basal cell carcinoma, but its efficacy is limited by reduced photosensitiser penetration and limited depth of light activation. These problems represent the main obstacle in the treatment of nodular and thicker lesions. Laser-assisted photodynamic therapy was developed to overcome these limitations through a preliminary laser-induced vaporisation of tumour tissue. Compared with conventional PDT, which achieves high clearance rates in superficial BCCs but suboptimal results in nodular lesions, laser-assisted PDT offers a relevant theoretical advantage. Partial ablation or microperforation may enhance photosensitizer penetration into deeper tumour layers, potentially extending efficacy to selected nodular BCCs. However, direct head-to-head comparisons in large, stratified cohorts remain limited [50,51].

One of the most relevant contributions is the prospective study by Ferrara and colleagues, who evaluated the combination of CO_2_ laser and conventional PDT in 32 patients with a total of 181 BCCs, both superficial and nodular. In the superficial ones, a fractionated CO_2_ was used, while in the nodular ones a continuous superpulsed CO_2_ was used, followed by application of MAL and red-light illumination. At three months, 100% clinical and dermoscopic clearance was observed, with a relapse-free rate of 97.2% during a mean follow-up of approximately 11 months. Adverse events were mild and the overall aesthetic outcome favourable [52].

An important methodological aspect is the comparison between continuous and fractionated modalities. A randomised intra-patient study compared continuous CO_2_ and fractionated CO_2_ as pre-treatment to PDT in superficial BCC and Bowen’s disease. Both strategies showed high efficacy at 12 months, with clearance above 90% and histological confirmation. The fractionated modality was better tolerated in terms of pain during illumination, with comparable safety profiles and aesthetic outcomes. This suggests that the choice of laser modality may be modulated according to patient size, site, and tolerability rather than marked differences in efficacy [53].

Clinical evidence supporting this approach also derives from a large prospective study including 286 patients with recurrent nodular basal cell carcinomas, of whom 194 were evaluable, reporting remarkably high clearance rates. Er:YAG laser-assisted PDT achieved a final clearance of approximately 99% with favourable aesthetic outcomes over a median follow-up of about 2 years, clearly outperforming PDT alone and Er:YAG monotherapy. However, the absence of long-term follow-up and systematic histological confirmation suggests that these results, while impressive, should be interpreted with caution [54].

A relevant evolution is the integration of non-invasive imaging. Reflectance confocal microscopy has been used to assess early response and long-term follow-up after laser-assisted PDT, particularly in facial nodular BCC treated successfully with superpulsed CO_2_ followed by PDT [55].

**Table 1:** Summary of laser modalities and main clinical outcomes.

Laser Source	Treated BCC Type	Typical Treatment Pattern	Main Clinical Outcome (Clearance Rate)	Adverse Events
CO_2_ Laser (Ablative) 10,600 nm [32,33,34,35,36,37]	Superficial, small nodular	Multiple passes, cleansing between passes. Peripheral clinical margin often treated.	Varies from 78.8% (vs. surgery) to 93.7% clearance (with intraoperative control). Long-term recurrence rate ~3% in selected cases.	Minor: erythema, crusting. Major: hypertrophic scar (1 case), hypopigmentation (1 case).
Pulsed Dye Laser (PDL) 595 nm [44,45]	Superficial, low-risk	1–5 sessions; fluence commonly ~6.5–8 J/cm^2^, pulse ~0.5–1.5 ms, spot ~7–10 mm, purpuric endpoint often used.	78.6% complete remission (vs 4.5% sham) at 6 months. 16/20 lesions with complete clinical response in a prospective study.	Minor: transient purpura, erythema, edoema; hypo/hyperpigmentation. Major: not reported.
Nd:YAG Laser 1064 nm [46]	Non-facial, < 2.1 cm	Often single session; spot ~5–6 mm, pulse ~7–10 ms, fluence commonly ~125–140 J/cm^2^; margins treated (~5 mm); no epidermal cooling in reported protocols.	Histological clearance around ~90% at short interval excision-based checks	Minor: erythema, edoema; hypo/hyperpigmentation; blistering. Major: not reported.
PDL + Nd:YAG Sequential Protocol [47]	BCCs smaller than 2 cm	Multiple sessions, PDL then Nd:YAG within the same visit.	Histology-verified clearance ~58% overall, higher (≈75%) in tumours <1 cm.	Minor: transient purpura, erythema, edoema; hypo/hyperpigmentation, blistering. Major: not reported.
Laser-Assisted PDT (CO_2_/Er:YAG + PDT) [52,53,54,55]	Superficial and selected nodular	Fractional or continuous modes before PDT; repeated sessions in some protocols.	High clearance rates (e.g., 97.2% relapse-free rate at 3 months with CO_2_ + PDT; ~99% clearance with Er:YAG + PDT at 2 years).	Minor: erythema, edoema, crusting, hypo/hyperpigmentation. Major: not reported.

## 3. Literature Gaps

Despite growing interest in laser-based approaches, the available evidence is affected by several methodological and reporting gaps that limit comparability and clinical translation. The key gaps identified in the literature are summarised in Table 2 and further discussed in the following sections.

### 3.1. Heterogeneity in Tumour Subtypes

Most published studies do not adequately specify the histological subtype of treated basal cell carcinoma. Generic terms such as “basal cell carcinoma” are frequently used without distinguishing between histological variants. However, biological behaviour and therapeutic response differ substantially between superficial, nodular and infiltrative or morpheiform BCC. This lack of detail significantly limits the interpretability of the results and reduces comparability between studies.

A recent systematic review highlighted this as one of the main limitations of the available literature. Only a minority of the studies analysed clearly report the histological subtype, while a significant proportion use generic terminology or provide no histological information at all [12,56,57].

The absence of histological stratification prevents the development of specific evidence-based recommendations. It is plausible that nodular BCCs require different laser parameters and therapeutic strategies than superficial forms, but the relative efficacy of different modalities cannot be defined without clear histological characterisation. For the same reason, clinical guidelines necessarily remain generic.

Future studies should systematically include detailed histological classification, with pre-treatment diagnosis based on biopsy or at least non-invasive imaging. Only through rigorous standardisation will it be possible to improve the quality of evidence and develop subtype-specific therapeutic indications.

### 3.2. Lack of Tumour Size Specification

Tumour size is often inaccurately reported in studies on laser treatment of basal cell carcinoma. Generic definitions such as small or medium-sized lesions are common, although size is a relevant prognostic factor. In fact, tumours smaller than 1 cm show higher clearance rates and a lower risk of recurrence than larger lesions.

Laser response is clearly size-dependent. Smaller lesions allow for a more uniform distribution of energy and a higher probability of complete eradication, while larger tumours may show areas of incomplete treatment. Available systematic reviews confirm that only a minority of studies report sufficiently precise size measurements, limiting the identification of clinically useful thresholds and the definition of reliable indications [12,55].

To improve the quality of the evidence, future studies should adopt a standardised measurement of tumour size based on pre-treatment clinical maximum diameter and categorisation into predefined size classes.

### 3.3. Methodological Heterogeneity

Methodological heterogeneity between studies represents a major barrier to the synthesis of evidence on laser therapy in basal cell carcinoma. Laser parameters vary substantially, even when the same devices are used, with differences in fluence, spot size, pulse duration, and cooling, limiting reproducibility and the definition of optimal settings. Treatment protocols are also heterogeneous, ranging from single-session approaches to multiple cycles with variable intervals, often without a clear rationale for session number or retreatment criteria, leaving treatment strategies largely empirical and centre-dependent. Endpoints further contribute to variability, as some studies rely on clinical clearance, while others require histological confirmation, frequently assessed at non-uniform time points. In this context, the available evidence was addressed through a critical, modality-specific discussion, underscoring the need for shared minimum criteria for technical reporting and more homogeneous outcome measures [56].

### 3.4. Limited Follow-Up Duration

The duration of follow-up represents one of the most significant limitations in the literature on laser therapy of basal cell carcinoma. In most studies, follow-up focuses on 12–24 months. This interval is often insufficient for a real evaluation. BCC may in fact recur even years after initial treatment, and the absence of data beyond 5 years introduces a bias that tends to overestimate long-term cure.

Only a small proportion of studies report follow-ups beyond 3 years. Exceptions exist, but they remain limited and often refer to specific contexts or techniques that do not fully overlap. Consequently, the risk of underestimating late recurrences is real, especially when the evaluation is based only on short-term clinical endpoints.

To make the evidence more credible, future studies should include longer follow-ups, ideally at least after 5 years, with structured surveillance and predefined timepoints. Prospective multicentre registries could be the most realistic tool to obtain efficacy and safety data truly comparable with oncology standards [12,57,58,59].

## 4. Future Perspectives and Conclusions

Based on the gaps that have emerged, future perspectives should shift from heterogeneous case series to more focused and comparable studies. There is a need for RCTs designed on well-defined populations, prioritising superficial or small nodular BCCs, those smaller than 1–1.5 cm and those located in low-risk areas, comparing laser therapy with standard surgery and adopting uniform parameters and outcomes. From a clinical perspective, laser-based treatments should be considered only in carefully selected patients with low-risk BCCs, while high-risk tumours and lesions in critical anatomical sites should continue to be managed with surgery and histological margin control.

Non-invasive imaging, in particular LC-OCT and RCM, is expected to play a central role in patient selection, early monitoring, and follow-up, reducing the risk of incomplete clearance that limits the credibility of destructive approaches today [23,60].

A still little explored area concerns combination therapies, which appear to be among the most promising strategies in the context of laser therapy [61,62]. Not only laser combined with topical drugs or laser-assisted PDT, but also combinations between different sources, such as CO_2_ and PDL, with or without PDT or topical treatments, many of which have not yet been systematically evaluated. Identifying which combinations provide the best balance between clearance, tolerability, and aesthetic outcome is a key step before extending clinical indications.

Emerging tools such as high-frequency ultrasound may assist pre-treatment planning by estimating tumour depth, while medical thermography could enable real-time monitoring of thermal diffusion during laser ablation. However, clinical evidence supporting these applications in laser-treated BCC remains preliminary [63].

In conclusion, laser therapy in BCC is an evolving field, with concrete advantages in terms of minimal invasiveness and cosmetic outcome but still limited by methodological heterogeneity, incomplete stratification, and short follow-up. With standardised protocols, objective controls, and a focus on non-aggressive tumours in low-risk sites, it could find a more defined clinical space within the current guidelines, also helping to reduce the surgical burden of operating theatres without compromising oncological safety [6,7].

## 5. Limitations

This work has several limitations related to its narrative design. The selection of studies was guided by clinical relevance and the author’s interpretation rather than by predefined systematic criteria, with a potential risk of selection bias. Critical reflections and future perspectives thus reflect the judgement of the authors and the articles considered rather than a formal quantitative synthesis. The absence of a meta-analysis limits the direct comparison between different laser modalities and standard surgery but is consistent with the aim of the work, which is oriented towards a critical and clinically applicable reading of the literature. These limitations were addressed through a critical discussion of study design, endpoints, and follow-up within the relevant sections of the manuscript.

## Figures and Tables

**Table 2:** Major gaps in the current literature on laser treatment for BCC.

Literature Gap	Description of the Limitation	Standard for Future Studies
Heterogeneity in Tumour Subtypes	Frequent use of generic terms (“basal cell carcinoma”) without distinguishing subtypes (superficial vs. nodular vs. infiltrative).	Mandatory histologic stratification and subgroup reporting.
Lack of Tumour Size Specification	Lack of standardised reporting; vague definitions (“small/medium”) instead of precise metric cut-offs.	Predefined size categories (e.g., <10 mm, 10–15 mm, >15 mm) and separate outcomes.
Methodological Heterogeneity	High variability in laser parameters (fluence, pulse duration, cooling) and treatment protocols (single vs. multiple sessions, interval timing).	Standardised, fully reproducible protocols with complete parameter reporting and predefined treatment algorithms.
Limited Follow-Up Duration	Most studies report outcomes at 12–24 months; data exceeding 3–5 years are rare.	Standardised follow-up monitoring with predefined recurrence criteria and minimum long-term observation targets.

## Data Availability

The original contributions presented in this study are included in the article. Further inquiries can be directed to the corresponding author.
